# Early Emergence and Selection of a SIV-LTR C/EBP Site Variant in SIV-Infected Macaques That Increases Virus Infectivity

**DOI:** 10.1371/journal.pone.0042801

**Published:** 2012-08-27

**Authors:** Shruthi Ravimohan, Lucio Gama, Elizabeth L. Engle, M. Christine Zink, Janice E. Clements

**Affiliations:** 1 Division of Infectious Diseases, University of Pennsylvania Perelman School of Medicine, Philadelphia, Pennsylvania, United States of America; 2 Department of Molecular and Comparative Pathobiology, Johns Hopkins University School of Medicine, Baltimore, Maryland, United States of America; 3 Department of Neurology, Johns Hopkins University School of Medicine, Baltimore, Maryland, United States of America; 4 Department of Pathology, Johns Hopkins University School of Medicine, Baltimore, Maryland, United States of America; Harvard Medical School, United States of America

## Abstract

CCAAT/enhancer binding protein (C/EBP)β, and C/EBP binding sites in the HIV/SIV- long terminal repeat (LTR) are crucial for regulating transcription and for IFNβ-mediated suppression of virus replication in macrophages, the predominant source of productive virus replication in the brain. We investigated sequence variation within the SIV-LTR C/EBP sites that may be under selective pressure *in vivo* and therefore associated with disease progression. Using the SIV-macaque model, we examined viral LTR sequences derived from the spleen, a site of macrophage and lymphocyte infection, and the brain from macaques euthanized at 10, 21, 42, 48 and 84 days postinoculation (p.i.). A dominant variant, DS1C/A, containing an adenine-to-guanine substitution and a linked cytosine-to-adenine substitution in the downstream (DS1) C/EBP site, was detected in the spleen at 10 days p.i. The DS1C/A genotype was not detected in the brain until 42 days p.i., after which it was the predominant replicating genotype in both brain and spleen. Functional characterization of the DS1C/A containing SIV showed increased infectivity with or without IFNβ treatment over the wild-type virus, SIV/17E-Fr. The DS1C/A C/EBP site had higher affinity for both protein isoforms of C/EBPβ compared to the wild-type DS1 C/EBP site. Cytokine expression in spleen compared to brain implicated IFNβ and IL-6 responses as part of the selective pressures contributing to emergence of the DS1C/A genotype *in vivo*. These studies demonstrate selective replication of virus containing the DS1C/A genotype that either emerges very early in spleen and spreads to the brain, or evolves independently in the brain when IFNβ and IL-6 levels are similar to that found in spleen earlier in infection.

## Introduction

Invasion of human immunodeficiency virus (HIV-1) and simian immunodeficiency virus (SIV) into the central nervous system (CNS) takes place during acute infection [1], [2], [3], but the development of HIV-associated neurocognitive disorders (HAND) does not occur until late in the disease process [4], [5]. Although highly active antiretroviral therapy (HAART) of HIV-1- infected individuals has significantly reduced the incidence of HIV-1 associated dementia (HAD), the prevalence of HAND continues to rise as the number of chronically infected HIV-1 patients increases [6], [7], [8], [9]. During HIV and SIV infection, virus evolution is a dynamic process driven by selective pressures, including immune responses and virus adaptation. Viral variants that have selective advantages for replication in the CNS may evolve very early in infection in the periphery, or may develop via independent evolution in the brain. Thus, an understanding of virus evolution and host factors that govern this process is necessary to develop novel therapies that curtail the development of highly pathogenic viral variants early in infection, thereby preventing infection of the brain, a viral reservoir that is difficult to target.

Although selective pressures exerted by the adaptive immune system on various viral genes such as *gag*, *pol,* and *env* (reviewed in [10], [11]) have been extensively researched, little is known about selection mediated by innate immune responses such as interferon (IFN) β. SIV and HIV infection trigger innate immune responses coordinated by type I IFNs, particularly IFNβ, the predominant and first type I IFN to be expressed in the brain [1], [12], [13]. IFNβ inhibits HIV/SIV replication in macrophages inducing expression of the truncated, dominant-negative isoform of the CCAAT/enhancer binding protein (C/EBP) β, also referred to as liver-enriched transcriptional inhibitory protein or LIP [13], [14], [15], [16], [17]. Down-modulation of long terminal repeat (LTR) activity by IFNβ has been demonstrated to be mediated through two C/EBP sites found within the SIV-LTR, specifically due to their high binding affinity for LIP [18].

The evolution of transcription binding sites found within the HIV/SIV-LTR, which are crucial for mediating transcription and viral replication, has not been thoroughly investigated. The regulation of HIV-1 transcription and replication in macrophages is mediated primarily by the two isoforms of C/EBPβ, the liver-enriched transcriptional activator protein (LAP) and LIP translated from the second and third in-frame AUG, respectively, and in these cells at least one functional C/EBP binding site within the HIV-1 LTR is necessary for basal level transcription and replication [19], [20], [21]. C/EBPβ has also been implicated in regulating LTR activity and virus replication in CD4^+^ T cells [22]. We have recently defined the functional roles of two C/EBP sites, JC1 (−100 bp, with respect to the transcription start site) and DS1 (+134 bp) C/EBP sites, in the SIV-LTR core promoter, that are crucial for basal transcription and productive virus replication, respectively [18]. Sequence variation in the HIV-1 C/EBP site and their correlation with disease progression have been examined in a few studies [23], [24], [25]. However, these studies were limited to the analyses of postmortem autopsy samples, making it impossible to identify the virus that infected the individuals, or track the evolution of viral variants simultaneously in the periphery and the brain through disease progression.

Based on these observations we hypothesized that the C/EBP sites within the core promoter of the SIV-LTR (−236 to +154 bp) are under selection [26], [27]. Therefore, we examined sequence variation *in vivo* in this region and investigated whether these sites were under different selective pressures in the periphery versus the brain at different stages of SIV disease progression. We used the spleen to investigate sequence variation in a peripheral tissue. However, as the infection of splenic macrophages has not been well characterized until now, we examined for the first time SIV infection of these cells, including virus replication and expression of cytokines in the spleen within our macaque model.

Here we used an established SIV macaque model for HIV/AIDS and CNS disease developed in our laboratory, where pigtailed macaques (*Macaca nemestrina*) are co-inoculated with SIV/17E-Fr, a macrophage-tropic neurovirulent recombinant virus clone that infects macrophages in the periphery and the CNS, and SIV/DeltaB670, an immunosuppressive swarm that causes more rapid decline in the CD4+ lymphocytes in the peripheral blood and tissues [1], [28], [29], [30], [31], [32]. Based on sequence analysis of the SIV-LTR, we identified an adenine-to-guanine substitution at position +123 bp and a linked cytosine-to-adenine substitution at position +146 bp within the DS1 C/EBP site. This genotype, termed DS1C/A, was detected early in infection (10–21 days p.i.) in the spleen, but only at 42 days p.i. in the brain, and was the predominant replicating genotype in both the brain and the spleen thereafter. Functional analyses indicated that SIV/17E-Fr containing the DS1C/A substitution had higher infectivity compared to the wild-type virus. Further, while IFNβ suppressed activity of both wild-type and DS1C/A containing virus, the DS1C/A virus sustained a higher level of infectivity over wild-type. Competition electrophoretic mobility shift assays (EMSA) demonstrated a higher affinity of the DS1C/A site for both isoforms of C/EBPβ, LAP and LIP, compared to wild-type DS1 C/EBP site. We also examined the cytokine environment and the presence of SIV infected macrophages and T-lymphocytes in the spleen. These data, in conjunction, with previous characterization of the brain [1], [13], [17] provided a mechanistic link between the selective pressures present *in vivo* during acute infection and later in disease that in part may contribute to the evolution and selection of the DS1C/A variant in the spleen and the brain.

## Results

Our previous studies have demonstrated the critical roles of C/EBP sites, JC1 and DS1, in regulating SIV-LTR transcriptional activity [18]. Regulation of the SIV and HIV LTRs by C/EBPβ has been demonstrated in both CD4+ lymphocytes and macrophages [21], [22]. In this study we examined whether the SIV-LTR C/EBP sites were under selective pressure in the periphery (spleen) and the brain of SIV-infected macaques through disease progression.

**Figure 1:**
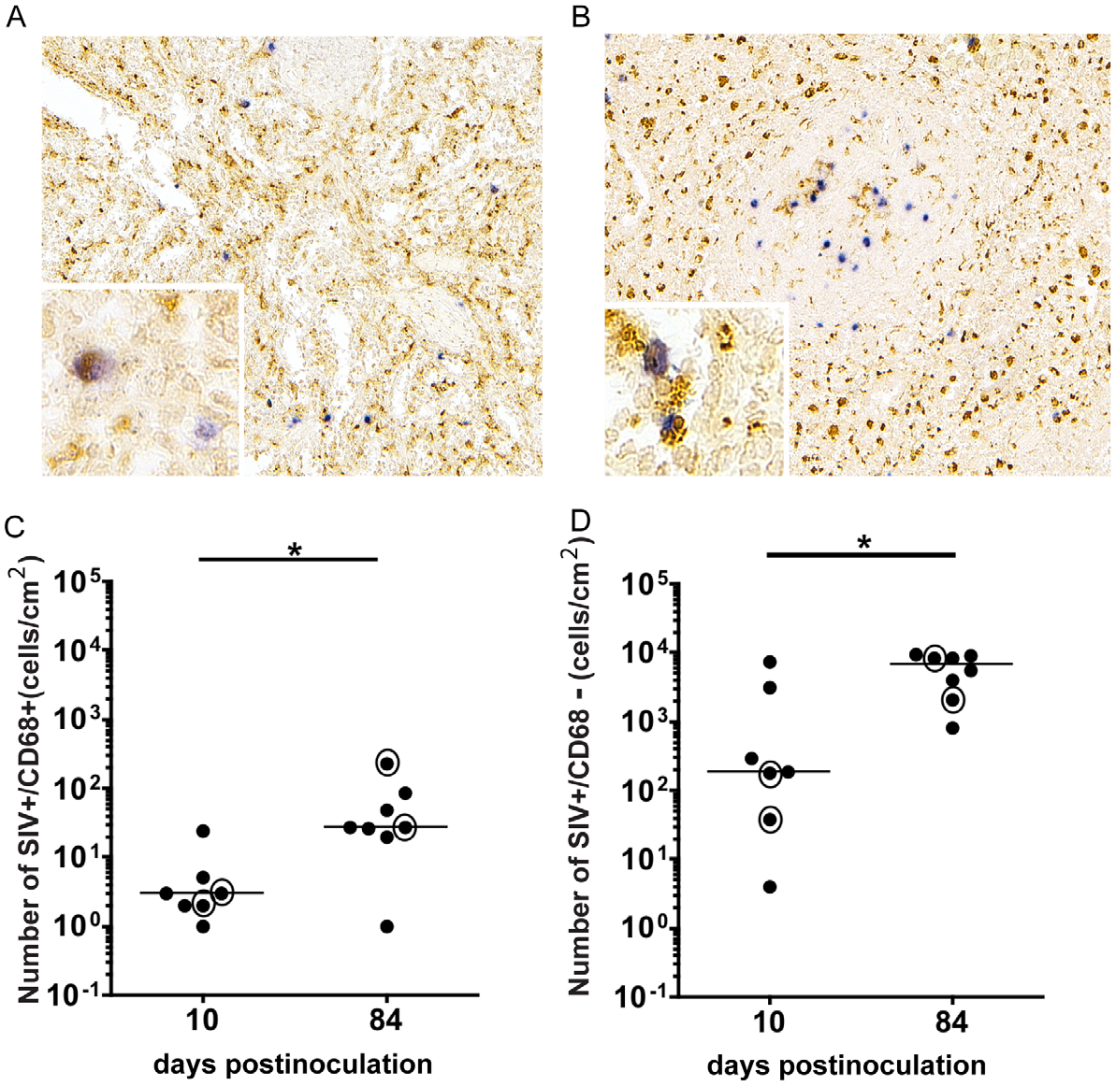
SIV infected macrophages in the spleen of SIV-infected macaques. Double immunohistochemistry/*in situ* hybridization labeled spleen from SIV-infected macaques euthanized at (A) 10 and (B) 84 days p.i. Brown precipitate indicates the macrophage marker CD68 and blue precipitate indicates SIV RNA. Insets show higher magnification of the section with SIV+/CD68+ macrophages. Number of double labeled SIV+/CD68+ macrophage cells/cm^2^ (C) and number of SIV+/CD68− cells/cm^2^ (D) in the spleen at 10 (N = 7) and 84 days p.i. (N = 8) were quantified. Medians are represented by black bars. Circles around dots denote animals for which sequencing was conducted. *P* values calculated by Mann Whitney Test (*p<0.05).

The spleen is recognized as a site of CD4^+^ T cell infection, but also contains macrophages, which are known to be targets of HIV and SIV infection. However, there are very few studies examining infection of macrophage lineage cells in the spleen [33], [34], [35]. To investigate the extent and timing of macrophage infection at this site, we examined the spleen of SIV-infected macaques during acute infection (10 days p.i.) and terminal disease (84 days p.i.). We employed immunohistochemistry to identify macrophages with the macrophage marker CD68, followed by *in situ* hybridization (ISH) for SIV RNA. SIV-infected macrophages (double labeled: SIV+/CD68+) were found in multiple sections of the red pulp, at 10 and 84 days p.i. (Figure 1A and B, respectively; [36]). Quantitation of SIV-infected, CD68 positive macrophages in the spleen revealed that there was a median of 3 SIV+/CD68 + cells/cm^2^ at day 10 p.i., which substantially increased to 27 cells/cm^2^ at day 84 p.i. (p<0.05; Figure 1C). However, this is a minimum estimate of the number of infected macrophages as there are other macrophage populations that do not express CD68. The number of SIV+ only cells, identified as SIV+/CD68− cells, representing predominantly lymphocytes as well as CD68 negative macrophages, were quantitated in the spleen at both time points (Figure 1D). At 10 days p.i., there was a median of 183 SIV+/CD68− cells/cm^2^, which significantly increased to 6911 cells/cm^2^ (p<0.05). The number of SIV+/CD68+and SIV+/CD68− cells/cm^2^ in each animal are provided in Table S1. In addition to the spleen being a secondary lymphoid organ, the presence of both SIV infected splenic macrophages and T-lymphocytes provided the rationale for using the spleen as a peripheral tissue for studying virus evolution in the periphery.

### Sequence Analysis of SIV/17E-Fr and SIV/DeltaB670 viral LTR Genotypes in Virus Stocks

We determined the LTR sequences of the virus used as inoculum (containing SIV/17E-Fr and SIV/DeltaB670) for infection of macaques to differentiate the original virus from genotypes that evolved during disease progression [37]. We analyzed 294 cloned sequences from the recombinant clone virus, SIV/17E-Fr, using 3 independently prepared stocks and 180 cloned sequences from the immunosuppressive swarm, SIV/DeltaB670, using 2 independently prepared virus stocks. We specifically sequenced the 3′ U3-R region because it becomes the 5′LTR of the proviral genome after integration of the viral DNA and thereby regulates transcription and replication. All sequence comparisons and alignments were made to a portion of SIV/17E-Fr LTR spanning –236 to +154 bp with respect to the transcription start site and the U3/R border (also referred to as the mid-LTR, mLTR) and was used as the reference sequence. Our sequence analysis focused on this region because it contains the minimal promoter (−225 to + 18b p) for regulating basal LTR activity in U937 promonocytic cells as well as the minimal region (+19 to + 149 bp) for transactivation [26], [27] and includes the JC1 (−100 bp) and DS1 (+134 bp) C/EBP sites that regulate basal transcription and viral replication [18].

**Figure 2:**
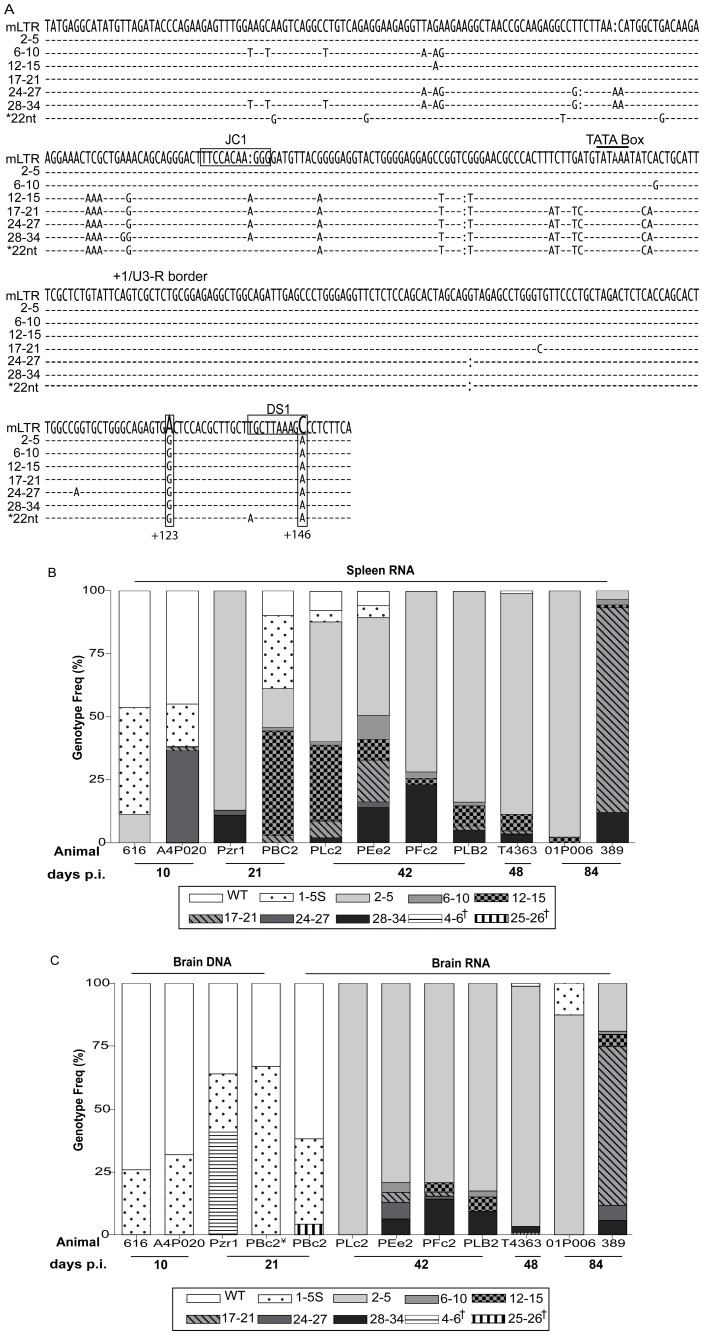
Sequence analysis of wild-type (WT) and variant LTRs detected in the spleen and brain. (A) Consensus sequence of variant LTR genotype groups were aligned to reference, wild-type (SIV/17E-Fr mLTR: −236 to −154 bp) sequence. Dashes (-) indicate conserved nucleotides; insertions and deletions are denoted by (:), horizontal rectangles highlight the JC1 and DS1 C/EBP sites. Vertical rectangles highlight +123 bp A/G and +146C/A substitutions within the DS1 C/EBP site. (*) 22nt variant found in 1 of 180 cloned sequences from the SIV/DeltaB670 swarm virus stock. (B) Frequency of LTR genotypes in spleen RNA from the indicated animals euthanized at 10, 21, 42, 48 and 84 days p.i. is depicted. Bars with gray/black background indicate the presence of DS1C/A in the LTRs sequenced. Bars with white background indicate absence of DS1C/A substitutions. (C) Genotype frequency of LTRs sequenced from brain RNA/DNA homogenates from each individual animal, as indicated. (¥) RNA derived LTR sequencing results for one 21 days p.i. animal, PBc2, are shown next to DNA derived LTR sequencing results from the same animal. (†) Indicates variant genotypes that are non-wild-type but lack the DS1C/A substitution.

LTR sequence analysis of the SIV/17E-Fr and SIV/DeltaB670 virus stocks revealed that the mLTR regions were indistinguishable between the two viruses with the exception of one clone out of 180 clones sequenced (genotype frequency <1%) from the SIV/DeltaB670 virus stock that had a cluster of 22-nucleotide (22nt) substitutions within this region (Figure 2A). As this genotype was detected at a very low frequency it was considered a minor genotype in the virus stock. This observation was in contrast to sequence variations seen in the *env* V1 region that serve as signature sequences to differentiate SIV/17E-Fr from the various genotypes present in the SIV/DeltaB670 swarm [28], [38], [39].

### Detection of Variant LTR Genotypes in the Spleen and Brain of SIV-infected Macaques

Viral genotypes present in the spleen and brain of SIV infected macaques euthanized at 10 (A4P020, 616), 21 (Pzr1, PBC2), 42 (PLc2, PEe2, PFc2, PLB2), 48 (T4363), and 84 (01P006, 389) days postinoculation (p.i.) were determined. We analyzed RNA whenever possible from the two tissues in order to determine LTR sequences in the actively replicating virus population. All the viral genotypes identified from the sequence analysis of the mLTR regions were compared to the wild-type, SIV/17E-Fr mLTR sequence used as reference (Figure 2A). For the analysis we divided the sequenced clones into groups based on the common subset of single nucleotide polymorphisms (SNPs) present in each clone; for example, clones that had two or five nucleotide substitutions when compared to the reference mLTR sequence were grouped together since the 2-SNP genotype was common to the changes found in the 5-SNP genotype. This group was classified as the 2–5 SNP variants (V) as it also included clones that had three or four other SNPs. Within the 2–5V group, the 2 SNPs at positions +123 bp and + 146 bp were always observed in all the clones with 3, 4, and 5 SNPs. Other substitutions in the 3, 4 and 5 SNP clones did not occur concurrently in the same clone, but several clones with these individual substitutions were detected. Using this scheme we classified the clones into six groups, namely, 2–5, 6–10, 12–15, 17–21, 24–27, and 28–34 V. Sequences that were grouped together were aligned to each other to determine the consensus sequence of the group, which was then aligned with the wild-type mLTR sequence, as shown in Figure 2A. All of these groups contained the 2 SNPs, at positions + 123 bp and + 146 bp. We also observed some clones that had wild-type genotype with one to five sporadic nucleotide substitutions (1–5 S) that did not recur consistently. These 1–5 S non-recurring substitutions could be due to the highly error-prone SIV reverse transcriptase that contributes to genetic diversity, but a small proportion may also be due to PCR error since they were within the expected range for Taq polymerase mediated errors of 0.2–2×10^−4^/bp/clone/cycle of PCR [40], [41], [42], [43]. In the sequencing results that follow, we focus on the frequency of the wild-type and 2–5V genotype group as they were the predominant genotypes in the spleen and brain. Details of genotype frequency for all other variants are in Table 1 and Figures 2B and C.

**Table 1:** Frequency of LTR genotypes in spleen and brain of SIV-infected macaques.

Tissue:	Genotype:	WT	1–5S	2–5	6–10	12–15	17–21	24–27	28–34	25–26†	4–6†	total # of clones
**Spleen**	**days p.i.**	**Genotype Frequency (%)**										
**RNA**	**10**	46	31	6			<1	17*				173
	**21**	5	16	48	<1	22	2	<1	5			135
	**42**	3	2	61	4	13	6	<1	10			348
	**48–84**	<1		62	<1	3	29	5				269
**Brain**												
**DNA**	**10**	71	29									188
	**21**	34	48								17	158
**RNA**	**21** Ŧ	62	34							4		94
	**42**			84	2	4		2	8			322
	**48–84**	<1	4	65		2	24	2	3			275

Summary of genotype frequencies (%) and total number of clones sequenced from spleen and brain at 10 (616, A4P020), 21 (Pzr1, PBc2), 42 (PLc2, PEe2, PFc2, PLB2), and 48–84 (T4363, 01P006, 389) days postinoculation (p.i.). The frequencies and total number of clones sequenced reflects those as shown in Figure 2B and C.

† Indicates variant genotype with the absence of both the +123A/G and +146C/A substitution within the DS1 C/EBP site.

* Indicates the absence of +123A/G but the presence of +146C/A (DS1C/A) substitution.

‡ Genotype frequency from analysis of brain RNA from macaques PBc2 euthanized at 21 days p.i.

### Early Appearance of the 2–5V Viral Genotype in Spleen

RNA from spleen of SIV-infected macaques listed above was used to track the frequency of variant viral genotypes actively replicating in the periphery (Figure 2B) and in brain (Figure 2C). Sequence analysis of clones from two animals euthanized at 10 days p.i. (616 and A4P020) demonstrated the presence of wild-type genotype at a frequency of 46% of the total clones sequenced from the two animals (Table 1). In these animals, wild-type LTR sequences with 1–5 non-recurring substitutions were detected at a frequency of 31%. The 2–5V genotype was detected in the spleen at a frequency of 11% in macaque 616 but not in macaque A4P020 (Figure 2B), thereby representing 6% of total 173 clones sequenced from both animals (Table 1).

Sequence analysis of spleen RNA at 21 days p.i. from animals Pzr1 and PBc2 showed a decrease in the frequency of wild-type genotype (5%) compared to the frequency at 10 days p.i. (46%). LTR wild-type sequences with 1–5 non-recurring substitutions also decreased in frequency from 31% at 10 days p.i. to 16% at 21 days p.i. However, the 2–5V genotype was found in 48% of the 135 total sequenced clones and present in all animals. Various other genotypes were also detected at this time point, all of which contained the 2-SNPs at positions + 123 bp and + 146 bp (Figure 2B and Table 1).

In the RNA from spleen of animals euthanized at 42 days p.i., wild-type sequence was detected at a frequency of only 3%. The 2–5 V genotype was observed at a frequency of 61% of the total sequenced clones from four animals (PLc2, PEe2 PFc2, and PLB2). At the terminal stages of disease (48–84 days p.i.), the frequency of the viral variants detected in the spleen RNA was similar to that observed at 42 days p.i. The frequency of wild-type LTR genotype in the periphery had dropped to <1%, while the 2–5 V genotype comprised 62% of the clone sequenced from the three animals (Table 1). Thus, between 42 and 48–84 days p.i., the wild-type LTR containing virus had become a minor frequency of the replicating genotypes, while the 2–5 V genotype represented 57% of the clones.

### Detection of Variant Genotypes in Brain after their Emergence in Spleen

In contrast to the spleen, early emergence of variant LTR genotypes was not observed in the brain of animals at 10 days p.i. (616, A4P020) and 21 days p.i. (Pzr1, PBc2), in which the genotypes detected consisted predominantly of wild-type LTR sequence (Figure 2C). In some animals, namely A4P020, 616 and Pzr1, sequences from the genomic viral DNA had to be used for analysis as the copies of viral RNA in brain of these animals were below the limit for cDNA synthesis and subsequent PCR amplification. For a thorough analysis we sequenced LTRs from brain DNA of all other animals (Figure S1).

Based on analysis of DNA sequences at 10 days p.i., 71% of the LTRs cloned from animals 616 and A4P020 were wild-type genotype and 29% had wild-type sequence with 1–5 S non-recurring substitutions (Table 1 and Figure 2C). Genotypes identified in the DNA represent both a collection of variants that have entered the brain throughout infection as well as those that are actively replicating at that time of analysis. Thus, the viral DNA sequences serve as an archive.

At 21 days p.i., the wild-type genotype was detected in 34% of brain DNA-derived LTRs from two animals (Pzr1 and PBc2), while 48% had wild-type LTR sequence with 1–5 S non-recurring mutations. We were able to sequence LTRs from brain RNA of animal PBc2, euthanized at 21 days p.i. in addition to DNA from brain. In this animal the wild-type LTR genotype represented 62% of the RNA derived clones while 34% had wild-type LTR sequence with 1–5 non-recurring substitutions (Table 1). Variant LTR sequences were also found in brain at 21 days p.i.; however, at a low frequency (Figure 2C and Table 1).

### Selective Replication of Variant Viral Genotypes in Brain at 42 Days p.i

In the SIV-infected macaques studied here, virus replication in brain is controlled during acute infection but resurges by 42 days p.i. [1]. We found that the genotypic diversity of virus replicating in brain reflected by analysis of RNA of four animals euthanized at 42 days p.i. (PLc2, PEe2, PFc2, and PLB2), increased and the wild-type LTR containing viruses were no longer detected. At this time, in contrast to the viral sequences in brain RNA, the wild-type genotype was detected in brain DNA at a frequency of 14% (Figure S1). This result indicated that the virus with the wild-type genotype had previously seeded the brain in these animals, but was no longer the predominant genotype in replicating viruses at 42 days p.i., the early stage of viral resurgence in the brain.

Furthermore, at 42 days p.i., the frequency of variant genotypes increased in the RNA and DNA from brain (Figure S1). Specifically, the 2–5 V genotype was found at a frequency of 84% in brain RNA (322 clones; Table 1) and 68% in brain DNA (591 clones; Figure S1).

At terminal stages of disease between 48 (T4363) and 84 (01P006, 389) days p.i., the wild-type genotype was detected at a frequency of <1% in the brain RNA (Table 1) and at a frequency of 2% in the DNA (Figure S1). The 2–5 V genotype was detected at a frequency of 65% in brain RNA (275 clones; Table 1) and 90% in brain DNA (286 clones; Figure S1) in three animals.

The aggregate results from animals sacrificed between 42 and 48–84 days p.i., demonstrated a shift from replication of virus with the wild-type mLTR genotype (<1%) to the 2–5 V genotype that comprised 75% of the total 597 clones sequenced from brain RNA. This result suggested that the 2–5 V genotype was the predominant genotype at late stage disease in brain while viruses with the wild-type LTR represented a minor component of the replicating viral genotypes, similar to the genotypes detected in spleen.

### DS1 C/EBP Site Variants are not Detected Early in Infection in the Brain, but Increase in Frequency by 42 Days p.i

**Figure 3:**
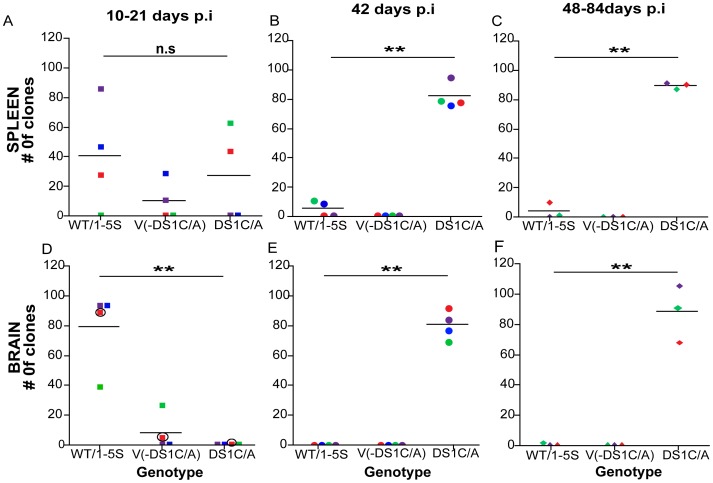
Change in occurrence of variant genotypes through disease progression in the spleen and brain. The number of clones with LTR sequences containing wild-type and 1–5 non-recurring substitutions (WT/1–5S) were pooled and compared to the total number of variants lacking the DS1C/A [V-(DS1C/A)] and to the total number of variants containing the +123A/G and +146C/A (DS1C/A) substitutions, at 10–21 days p.i. in the spleen (A) and brain (D, circles around the square represents RNA sequences); at 42 days p.i. in the spleen (B) and brain (E); and at 48–84 days p.i. in the spleen (C) and brain (F). Matched symbol and color represent the number of clones in the spleen and brain of the same animal at the indicated time postinoculation. Bars indicate mean number of clones sequenced per group. One-way ANOVA with Bonferroni post-tests were used to calculate *p*-values. The *p*-values shown in the figure correspond to the Bonferroni post-test comparing number of clones containing WT/1–5S to DS1C/A(**p<0.001; n.s: non-significant).

The 2–5 V genotype contained an adenine (A)-to-guanine (G) substitution at position +123 bp (+123A/G), 23 bp upstream of the DS1 C/EBP site and a cytosine (C)-to-adenine (A) substitution at position +146 bp (+146C/A), within the DS1 C/EBP site. Since the +123A/G and +146C/A substitutions occurred together in all variant genotypes: 6–10, 12–15, 17–21, 24–27 and 28–34 V (except in a single animal, A4P020, in which the 24–27 V genotype contained the +146C/A but lacked the +123A/G substitution) these two SNPs appear linked (Figure 2A) and therefore, are collectively referred to as the DS1C/A genotype. A comparison was conducted between the number of clones containing LTRs with wild-type or one to five non-recurring mutations (1–5 S), which were pooled together as WT/1–5S for analysis, to those clones lacking the DS1C/A genotype [V-(DS1C/A)] and those with the DS1C/A genotype, in the spleen and brain over time (Figure 3). Sequences analyzed in this way from the spleen revealed that between 10–21 days p.i. the number of clones with WT/1–5 S and DS1C/A genotypes did not significantly differ (Figure 3A). However, in animals infected for 42 and 48–84 days (Figures 3B and 3C), the DS1C/A containing genotypes increased significantly in the periphery compared to the wild-type virus (p<0.001), representing 97% of the total clones sequenced in animals infected for 42–84 days p.i.

In striking contrast, variants containing the DS1C/A genotype were not detected in RNA or DNA from brain of SIV-infected 10 and 21 days p.i. animals when compared to LTRs containing the WT/1–5S and V-(DS1C/A) (p<0.001; Figure 3D). The DS1C/A genotype was identified as the predominant replicating genotype for the first time at 42 days p.i. and continued to be the predominant genotype during terminal stages of disease, 48–84 days p.i. (p<0.001, Figures 3E and 3F). Viral genotypes containing the DS1C/A substitutions represented 98% of the RNA derived LTRs from a total of seven macaques analyzed at 42 (PLc2, PEe2, PFc2, and PLB2), 48 (T4363), and 84 (01P006 and 389) days p.i., similar to observations made in the spleen during late stages of disease.

### DS1C/A Variant Confers Increased Infectivity Despite Susceptibility to IFNβ Treatment

We have previously demonstrated that the JC1 and DS1 C/EBP site in the SIV-LTR are essential for basal transcription and productive viral replication in primary macrophages, respectively [18]. Our *in vivo* sequencing results demonstrate that mutations within the C/EBP site, specifically the DS1 site, emerged early in the spleen, but was not found in the brain until 42 and 48–84 days p.i. Additionally, as virus with the DS1C/A mutation predominates over virus containing wild-type LTR sequence after its emergence, the data suggests that this variant may have a growth advantage over the wild-type. These results prompted us to characterize the variant genotype in terms of virus replicative functions and the binding to C/EBPβ isoforms.

**Figure 4:**
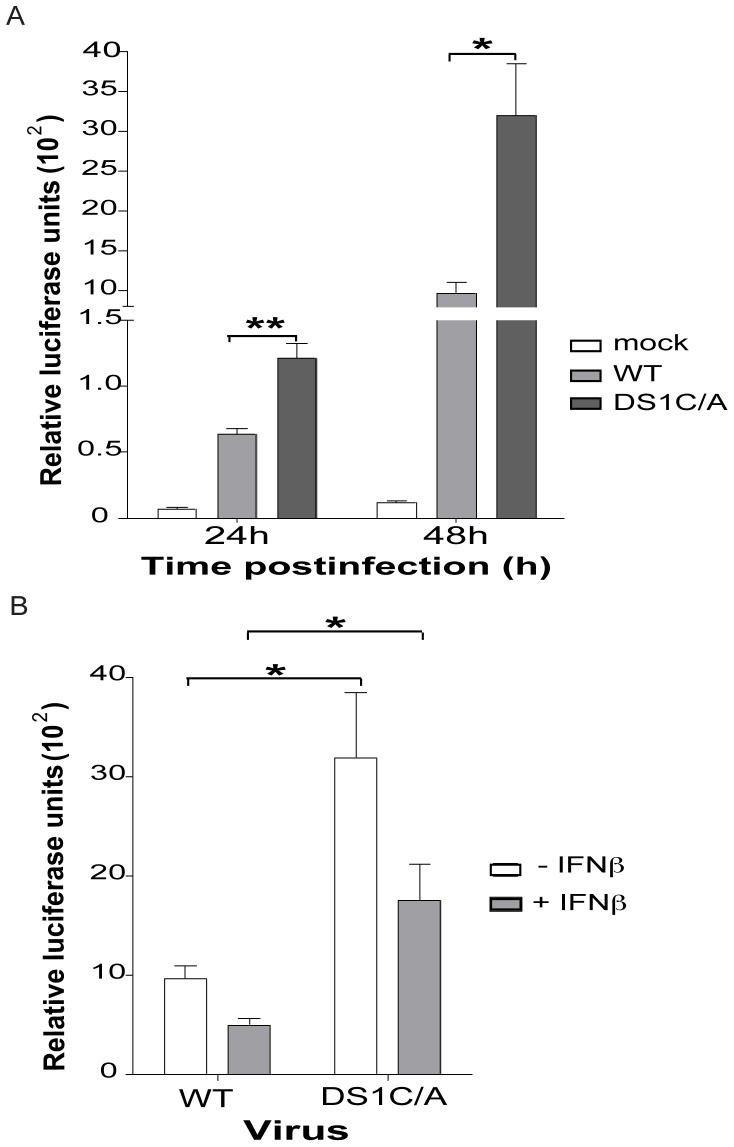
DS1C/A virus genotype confers a higher infectivity compared to wild-type (WT) virus despite IFNβ-meditated downregulation. LuSIV cells were infected with SIV/17E-Fr or SIV/17E-Fr virus containing the DS1C/A substitutions. (A) Luciferase assays was carried out at 24 and 48h post-infection (p.i.). (B) LuSIV cells were infected with SIV/17E-Fr or DS1C/A mutant virus followed by IFNβ treatment at 4 h p.i. Luciferase assays were carried out at 48 h after infection. Data is presented as mean ± S.E. from ≥ 3 independent experiments carried out in triplicate. *P*-values are calculated by Student’s two-sided paired t-test (*p<0.01; **p<0.001).

Site-directed mutagenesis was used to introduce the +123A/G and +146C/A nucleotide changes into the SIV/17E-Fr DNA, previously subcloned into the plasmid pUC19. Virus stocks were made in HEK293T cells using the wild-type SIV/17E-Fr and mutant-DS1C/A viral DNA. A Tat based luciferase reporter assay system was used to assess SIV replication in LuSIV cells, a modified CEMX174 cell line stably transfected with a portion of the SIVmac239 LTR cloned upstream of the firefly luciferase reporter gene [44]. Upon infection of these cells with HIV/SIV, the luciferase gene is expressed as a result of SIV-LTR transactivation by the viral HIV/SIV Tat protein [44]. Luciferase activity is directly correlated with virus infectivity [44]. In the LuSIV cells the DS1C/A mutant virus had a 1.9-fold (p<0.001) increased infectivity compared to SIV/17E-Fr at 24h postinfection after a single round of replication (Figure 4A). At 48h postinfection, after viral spread, the DS1C/A mutant had a 3-fold (p<0.01) increased infectivity over SIV/17E-Fr (Figure 4A). Similar results were obtained when these experiments were repeated using virus stock made in CEMX174 cells (data not shown).

IFNβ has been implicated in the suppression of acute SIV replication in the brain of infected macaques and shown to inhibit HIV/SIV replication in macrophages through the induction of the truncated, dominant-negative isoform of C/EBPβ, LIP [15], [16], [17], [45]. The DS1 C/EBP site in the SIV-LTR is particularly important for regulation by IFNβ [18]. The C-to-A substitution we identified in LTRs sequenced *in vivo* is within the DS1 C/EBP site. We investigated the functional relevance of the DS1C/A substitution as compared to wild-type DS1 C/EBP site, in the presence and absence of IFNβ, and whether such antiviral responses could explain why this genotype is undetectable in the brain early in infection (10–21 days p.i.) but emerges as a predominant genotype late in disease (42–84 days p.i.) when innate immune responses can no longer control virus replication [1]. Infection of LuSIV cells with the wild-type and DS1C/A mutant virus followed by IFNβ treatment at 4 h postinfection demonstrated that IFNβ downregulates LTR activity of both wild-type and DS1C/A genotype by 50% at 48 h postinfection (similar reduction was seen at 24 h postinfection with IFNβ treatment, data not shown). Nonetheless, the DS1C/A virus maintains 3-fold higher infectivity over wild-type even upon IFNβ-mediated downregulation (Figure 4B; p<0.01). Thus, the *in vivo*-identified nucleotide changes, +123A/G and the +146C/A within the DS1 C/EBP site, led to increased infectivity compared to wild-type virus although both were similarly susceptible to IFNβ regulation.

### DS1C/A Site has Higher Binding Affinity for LAP and LIP Compared to Wild-type DS1 C/EBP Site

The DS1 C/EBP site has been shown to bind both isoforms of C/EBPβ, LAP and LIP [18], [46]. Because the DS1C/A nucleotide substitution is within the DS1 C/EBP site, we assessed the binding of the two C/EBPβ isoforms to the DS1C/A site in comparison to the wild-type DS1 C/EBP site using electrophoretic mobility shift assays (EMSAs). The EMSAs demonstrated that, despite the nucleotide substitution, the DS1C/A site retained its ability to bind LAP and LIP (Figure S2).

**Figure 5:**
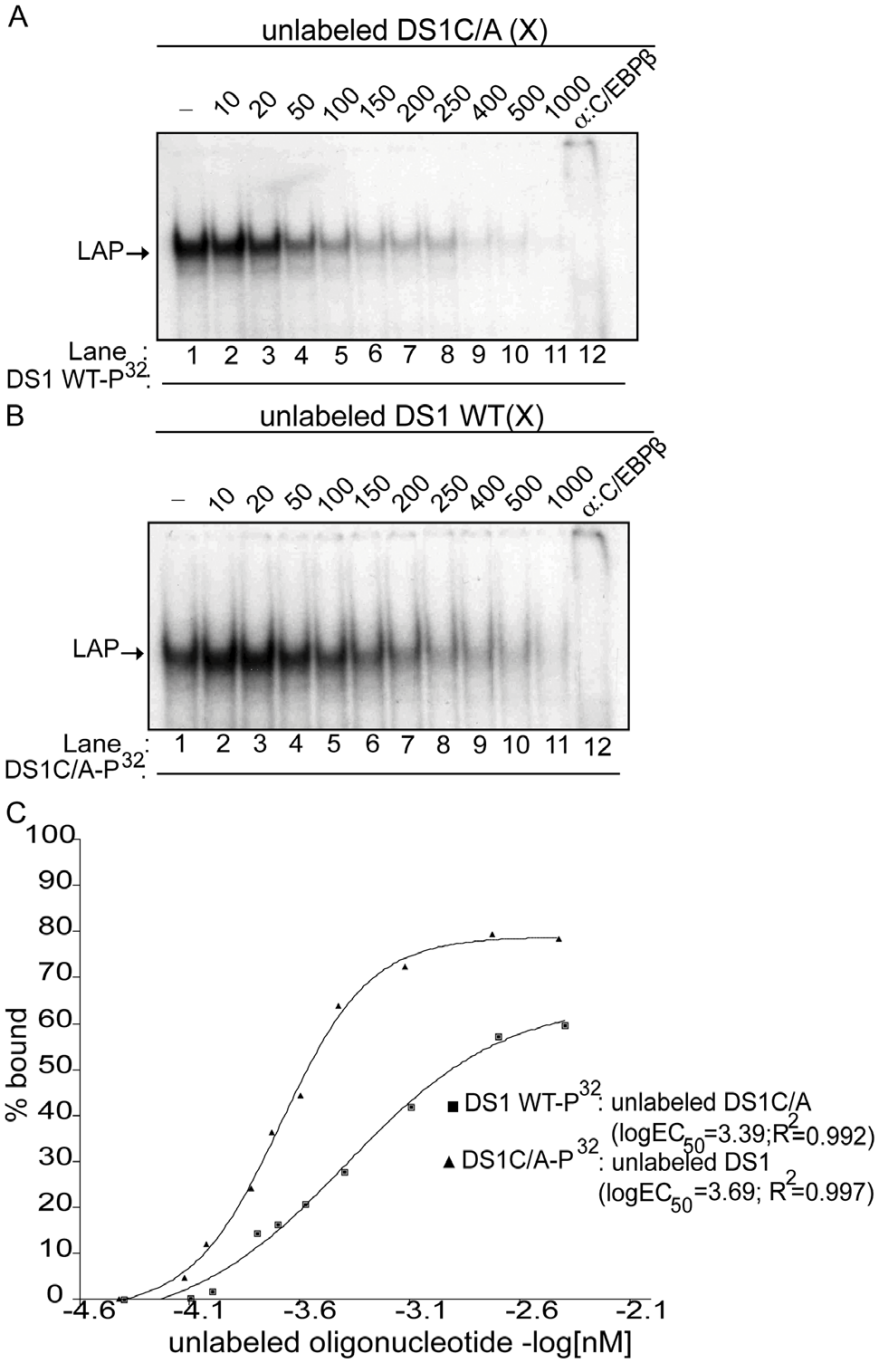
The DS1C/A site has a two-fold increased affinity for LAP compared to the DS1 wild-type C/EBP site. (A) Competition EMSA using nuclear extract obtained from HEK-293T cells transfected with the pCMV-LAP construct and incubated with labeled DS1 wild-type (WT) oligonucleotide alone (lane 1); 10 to 1000-fold molar excess (X) unlabeled DS1C/A oligonucleotide as indicated (lanes 2–11); or incubated with labeled DS1WT oligonucleotide and anti-C/EBPβ antibody (lane 12). (B) Competition EMSA using nuclear extract obtained from HEK-293T cells transfected with the pCMV-LAP construct and incubated with labeled DS1C/A oligonucleotide alone (lane 1); 10 to 1000-fold molar excess (X) unlabeled DS1WT oligonucleotide (lanes 2–11); or labeled DS1C/A oligonucleotide and anti-C/EBPβ antibody (lane 12). (C) Concentration of unlabeled oligonucleotide (-log[nM]) versus the percentage (%) of LAP bound to the labeled oligonucleotide, as determined by densitometric analysis of band intensities from the competitive EMSAs (A and B), was plotted. A non-linear regression curve was used to determine the K_D_ of LAP for the DS1 WT and DS1C/A sites from the logEC_50_ (half maximal protein binding) values as described in the Materials and Methods. Shown are representative EMSAs (n = 2).

**Figure 6:**
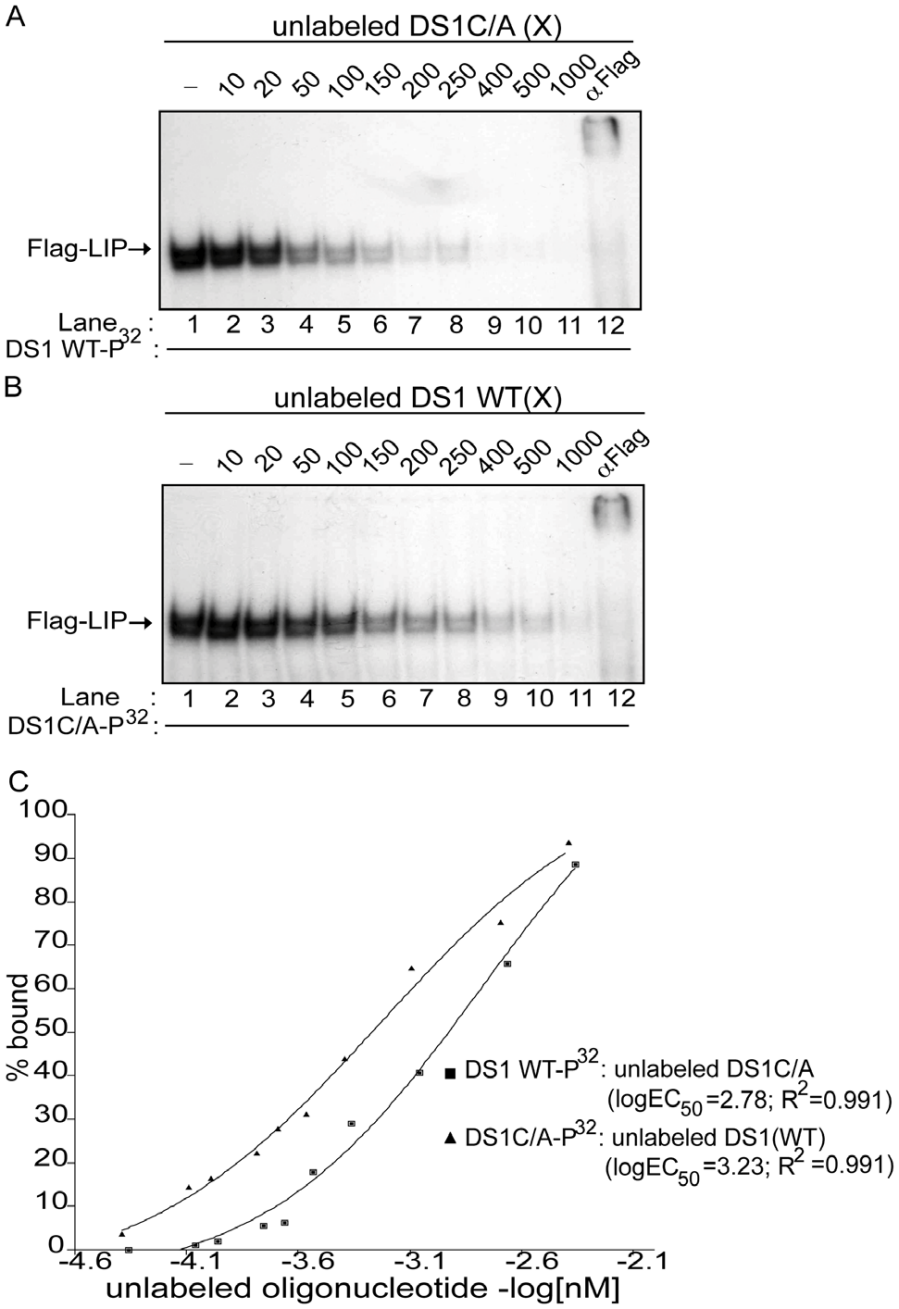
The DS1C/A site has a 3.5-fold increased affinity for LIP compared to the DS1 wild-type C/EBP site. (A) Competition EMSA using nuclear extract obtained from HEK-293T cells transfected with the pCMV-Flag-LIP construct and incubated with the labeled DS1 WT oligonucleotide alone (lane 1); 10 to 1000-fold molar excess (X) unlabeled DS1C/A oligonucleotide (lanes 2–11); or labeled DS1 WT oligonucleotide and anti-FLAG antibody (lane 12). (B) Competition EMSA using nuclear extract obtained from HEK-293T cells transfected with the pCMV-Flag-LIP construct and incubated with labeled DS1C/A oligonucleotide alone (lane 1); 10 to 1000-fold molar excess (X) unlabeled DS1 WT oligonucleotide (lanes 2–11); or labeled DS1C/A oligonucleotide and anti-FLAG antibody (lane 12). (C) Concentration of unlabeled oligonucleotide (-log[nM]) versus the percentage (%) of Flag-LIP bound to the labeled oligonucleotide, as determined by densitometric analysis of band intensities from the competitive EMSAs (A and B), was plotted. A non-linear regression curve was then used to determine K_D_ of Flag-LIP for the DS1C/A and DS1 WT C/EBP sites from the logEC_50_ (half maximal protein binding) values. Shown are representative EMSAs (n = 2).

We have previously shown using competition EMSAs that the affinity of LIP for the wild-type DS1 C/EBP site is 3.5-fold higher than that of LAP, which may contribute to the negative regulatory function of the wild-type DS1 C/EBP site with regard to SIV-LTR activity [18]. We therefore carried out competition EMSAs to determine whether the C-to-A substitution within the DS1 site alters the affinity of LAP (Figure 5) and/or LIP (Figure 6). LAP containing nuclear extracts were incubated with P^32^-labeled DS1 wild-type (WT; Figure 5A) or DS1C/A (Figure 5B) oligonucleotide in the presence of increasing concentrations of unlabeled DS1C/A (Figure 5A) or DS1 WT oligonucleotide (Figure 5B). Based on densitometric analysis of the band intensities (Figure 5C), the K_D_ of LAP for the DS1C/A and the wild-type DS1 C/EBP site were determined to be 24.8×10^−7^ M and 50.0×10^−7^ M (similar to previously published data [18]), respectively. This corresponded to an approximately two-fold increase in the affinity of LAP for the DS1C/A site as compared to the wild-type DS1 site (Table 2).

**Table 2:** Affinity of DS1C/A and DS1 C/EBP sites for C/EBPβ isoforms.

	C/EBP site K_D_ a (10^−7^M)		Relative affinity^b^ of DS1C/A
C/EBPβ isoform	DS1	DS1C/A	(fold increase)
LAP	50±2.0	24.8±0.4	2.0
Flag-LIP	17.5±0.9	5.1±1.3	3.5

a K_D_ - values were calculated from densitometric analysis of band intensities from competition EMSAs and plotting the concentration of unlabeled oligonucleotide (-log[nM]) versus the percentage (%) of LAP or LIP bound to the labeled oligonucleotide (FIG. 5C and 6C).

b Increase in relative affinity of LAP or LIP for the DS1C/A site compared to the wild-type DS1 C/EBP site was calculated using the equation: (DS1 K_D_)/(DS1C/A K_D_).

Similar competition EMSAs were carried out using Flag-tagged LIP-containing nuclear extracts (Figure 6) incubated with P^32^-labeled DS1 WT (Figure 6A) or DS1C/A (Figure 6B) oligonucleotide and competed with increasing concentrations of unlabeled DS1C/A (Figure 6A) or wild-type DS1 C/EBP (Figure 6B), as indicated. Based on the densitometric analysis (Figure 6C), the K_D_ of LIP for the DS1C/A and wild-type DS1 C/EBP sites were determined to be 5.1×10^−7^ M and 17.5×10^−7^ M, respectively. This difference in K_D_ corresponds to an approximately 3.5-fold increase in the affinity of LIP for the DS1C/A site over the wild-type DS1 C/EBP site. Furthermore, based on the above K_D_ values when comparing affinities of LIP and LAP for the DS1C/A site and wild-type DS1 site, LIP has a 5-fold higher affinity than LAP for the DS1C/A site compared to a 3-fold higher affinity of LIP over LAP for the wild-type DS1 site.

### Higher Viral Loads and Earlier Peak in IFNβ and IL-6 mRNA Expression During Acute and Early Infection in the Spleen Compared to the Brain

**Figure 7:**
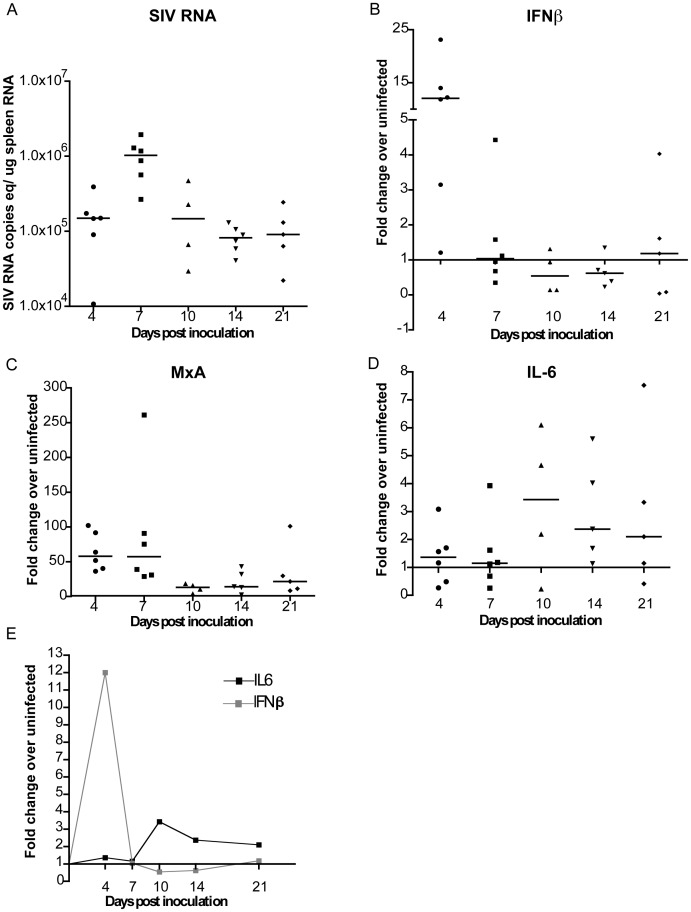
Characterization of the spleen in SIV-infected macaques during acute and early infection. RNA isolated from the spleen of uninfected and infected macaques euthanized at 4, 7, 10, 14, or 21 days postinoculation (p.i.) was used to quantitate (A) SIV RNA copies equivalents/ug spleen RNA as well as mRNA expression of (B) IFNβ, (C) MxA, and (D) IL-6 by quantitative RT-PCR. mRNA expression is represented as fold change over the average of three uninfected spleen mRNA, calculated by ΔΔCt. Black bars indicate medians for each time point. (E) Summarizes the medians of IFNβ and IL-6 mRNA levels shown in (B) and (D) at the indicated time points.

Virus evolution is a dynamic process influenced by several host and pathogen specific factors. We speculated that the cytokine environment, including innate immune responses that may act as selective pressures, could be different in the periphery and the brain; thereby, contributing to the earlier emergence of the DS1C/A genotype in the periphery compared to the brain. IFNβ regulates LIP levels and thus virus replication *in vitro* and *in vivo* in the brain during acute and late in infection [1], [14], [17]. Furthermore, IL-6 expression has been shown to regulate the expression of the activator isoform of C/EBPβ, LAP, which in turn induces IL-6 expression [47], [48], [49], [50], [51]. Changes in the levels of IL-6 mRNA expression in the brain through disease progression have been previously determined [1]. Thus, for direct comparisons to the brain, we determined the levels of virus replication and expression of IFNβ, MxA (IFNβ-induced gene) and IL-6 mRNA during acute and early infection in the spleen by quantitative real-time PCR (RT-PCR; Figure 7). Median levels of SIV RNA increased from 4 (1.5×10^5^ SIV RNA copies/µg spleen RNA) to 7 days p.i. (1.04×10^6^ SIV RNA copies/µg spleen RNA; Figure 7A) and peaked at 7 days p.i. Levels of virus replication were maintained in the spleen between 10–21 days p.i. In comparison, viral RNA levels in the brain were approximately 3 logs lower than in the spleen at 4 days p.i. and at 2 logs lower at 7 days p.i. (data previously published by Witwer et al. [1]). Furthermore, SIV RNA in the brain peaks between 10 and 14 days p.i. compared to 7 days p.i. in the spleen.

IFNβ (Figure 7B) and MxA (Figure 7C) mRNA levels were measured using RNA from the spleen of the same animals. IFNβ was induced by 12-fold over uninfected control at 4 days p.i. in the spleen, decreasing thereafter to levels comparable to uninfected at 7 days p.i. and remained low through 21 days p.i. Expression of MxA followed IFNβ expression.

IL-6 mRNA expression in the spleen was induced by only 1.3-fold above uninfected control at 4 days p.i. However, by 10 days p.i. IL-6 mRNA levels were induced 3.4-fold higher than uninfected animals (Figure 7D). Between 14 and 21 days p.i. IL-6 expression levels were maintained at 2.4- and 2.1-fold above uninfected control, respectively (Figure 7D).

In the spleen IFNβ levels peak earlier and were induced to higher levels (4 days p.i.) compared to the brain (7 days p.i.; [1]). While, the pattern of IL-6 induction was similar in both tissues following the decline of IFNβ in the spleen (Figure 7E) and in the brain [1].

## Discussion

The transcription factors of the C/EBPβ family and the C/EBP sites within the HIV-1 and SIV-LTR are critical for mediating virus replication in macrophages, which are the main source of productively replicating HIV/SIV in the brain [18], [21], [52], [53], [54], [55]. We have previously demonstrated that two C/EBP sites (JC1 and DS1) are essential for regulating SIV-LTR activity and virus replication, where mutations in the DS1 C/EBP site significantly disrupt virus replication in primary macrophages [18]. In this study we utilized a consistent and accelerated SIV macaque model for HIV/AIDS and CNS disease to investigate sequence variation within the core promoter region of the SIV-LTR [1], [26], [27], [31], [32], [56], [57]. Sequence analysis of virus derived from the spleen and brain from acute infection to late stage disease revealed that the downstream-1 (DS1) C/EBP site is, indeed, under selective pressure. We identified a variant, DS1C/A, with mutations in this C/EBP site that begins to replace the wild-type virus in spleen early in infection (10–21 days p.i.). This variant was found in brain only later in disease (42–84 days p.i.). Functional analyses of SIV containing the *in vivo* DS1C/A mutation indicated that this virus had increased infectivity compared to wild-type SIV/17E-Fr as well as higher affinity for both isoforms of C/EBPβ compared to the wild-type site. These data suggest that the selective advantage of SIV-DS1C/A *in vivo* in the spleen and brain is due to increased transcription. We also demonstrate, for the first time in our model, early replication of SIV in splenic macrophages, as well as the early selection of the DS1C/A genotype in this tissue. The selected mutation in the DS1 C/EBP site could have occurred in either T-lymphocytes or macrophages during acute infection in spleen; however, this variant is clearly selected for later in disease progression within the brain, where macrophages are the predominant productively infected cell type.

Functional analyses of SIV/17E-Fr containing the DS1C/A *in vivo* mutation demonstrated that this mutant virus had increased infectivity compared to wild-type SIV/17E-Fr virus. This increased infectivity was seen despite both viruses having similar susceptibility to IFNβ treatment. Examination of the binding affinity of both isoforms of C/EBPβ demonstrated that 1) the affinities of LAP (activating isoform) and LIP (inhibitory isoform) were higher for the variant DS1C/A C/EBP site compared to the wild-type DS1 C/EBP site and 2) that the DS1C/A mutation maintains a higher affinity for LIP compared to LAP. This higher affinity of LIP compared to LAP has been similarly observed for the canonical C/EBP site as well as for the wild-type JC1 and DS1 C/EBP sites [18], [58]. These results suggest that changes in the C/EBP binding sites that increase affinity for LIP also increase the affinity for LAP simultaneously; hence, the C/EBP site evolves to an overall higher affinity C/EBPβ binding site. Virus with such mutations would, therefore, have a selective advantage over wild-type as a result of the increased transcription and replication due to the increased affinity for LAP that this mutation confers.

Higher levels of replication and the cytokine environment found in the spleen during acute infection may act as selective pressures that contribute to the earlier emergence of the DS1C/A genotype in the spleen compared to the brain. We observed a 100-fold higher level of virus replication in the spleen compared to the brain between 7 and 10 days p.i.; thereby, increasing the probability that this mutation would occur during acute infection in the spleen. Both IFNβ and IL-6 have been shown to directly modulate levels of the two isoforms of C/EBP proteins, thereby regulating LTR activity and virus replication. IFNβ mediates induction of the LIP, while IL-6 not only increases HIV-1 virus replication in macrophages, but has also been implicated in inducing the expression of the activating isoform of C/EBPβ, LAP, [13], [14], [15], [16], [17], [49], [51], [59]. C/EBPβ expression in turn has been demonstrated to increase IL-6 expression creating a positive feedback loop [47], [48], [50]. Combining the functional characteristics of the DS1C/A mutant and IFNβ expression patterns in the spleen during acute infection when IFNβ mRNA levels peak at 4 days p.i., there may be a selective advantage for variant viruses with increased affinity for LIP that also inherently have a higher affinity for LAP. IFNβ mRNA levels are rapidly downregulated to uninfected control levels in the spleen by 7 days p.i. and is followed by increase in levels of IL-6 mRNA. At this time the DS1C/A variant may have greater replicative advantage over wild-type as a result of its higher affinity for LAP and in turn increased transcription. Thus the timing of expression of these cytokines seems crucial in the selection of the DS1C/A virus in the spleen.

IFNβ, LIP and LAP protein levels may explain the emergence of the DS1C/A genotype later in disease progression in the brain. We have previously shown that increase in IFNβ protein levels are accompanied by increase in LIP:LAP ratio during acute infection and are concurrent with a decline in virus replication in the brain [1], [17]. However, this transcriptional control of virus replication fails in many macaques by 42 days p.i., when viral recrudescence is accompanied by increase in proinflammatory cytokine expression such as IL-6 and TNF-α [1]. Additionally, there was a direct correlation in the levels of both SIV RNA and IL-6 mRNA with the ratio of LAP:LIP in the brain of SIV-infected macaques at 42 days p.i. [1]. Based on these observations, we hypothesize that during acute infection there is a constraint on replication of variants with the DS1C/A genotype in the brain mediated by higher levels of IFNβ and LIP:LAP ratio. However, as disease progresses and virus replication resurges in the brain at 42 days p.i., variants with the DS1C/A genotype are detected in the brain either because infected cells traffic from the periphery into the brain or because of independent co-evolution of this variant genotype in the brain when the rates of replication and IFNβ and IL-6 expression are comparable to that in the spleen during acute infection. Additionally, the SIV/17E-Fr is a macrophage-tropic neurovirulent, recombinant clone virus. Thus in the context of macrophages, which are also the predominant source of productive virus replication in the brain as well as IL-6, an advantageous environment is likely established where the IL-6 expression drives LAP expression; hence, the selection of SIV/17E-Fr virus with LTRs containing the DS1C/A C/EBP site mutation with increased binding affinity for LAP.

The presence of persistently infected macrophages in the spleen has been demonstrated in only one other study using a SHIV model after lymphocyte depletion [35]. However, we provide evidence for SIV-infected splenic macrophages and lymphocytes during acute infection as well as late in disease. This finding is important, as both cell types are potential sources of the viral variants we detected in the spleen. The DS1C/A variant is important in the context of both CD4^+^ T cells and macrophages, and of particular relevance to our studies, considering the early emergence of the DS1C/A genotype in the spleen. Despite the study by Henderson et al. that found that C/EBPβ mediated regulation is dispensable in PMA stimulated CD4^+^ T cells, Dumais et al. reported that C/EBP sites and C/EBPβ/CREB complexes are important in prostaglandin E_2_-dependent activation of HIV-1 LTR in CD4^+^ T cells [19], [22]. These observations suggest that C/EBPβ regulation of HIV-1 is not limited to macrophages.

The selective advantage conferred by the DS1C/A substitutions over the wild-type genotype is underscored by the fact that the DS1C/A substitutions were identified in all the variants groups: 2–5, 6–10, 12–15, 17–21, 24–27 and 28–34V. The 2–5V genotype, specifically, was the predominant genotype replicating in the brain and spleen, and contains the aforementioned C-to-A substitution in the DS1C/EBP site (DS1C/A). However, the JC1 C/EBP site, which we have demonstrated to be important in mediating basal level transcription, is completely conserved [18]. We have also shown that the JC1 C/EBP site has higher affinity for LAP compared to wild-type DS1C/EBP site and interestingly, the K_D_ of the JC1 C/EBP site is similar to that of the DS1C/A variant site [18]. Thus, the 2–5V genotype viral variant has two high affinity LAP binding sites, providing a mechanism for the increased replication of this viral variant compared to wild-type virus in the brain and spleen during disease development. This is pertinent during 7–21 days p.i. in the spleen and between 42–84 days p.i. in the brain when there is higher level of IL-6 expression compared to IFNβ, probably contributing to alterations in the ratio of LIP:LAP and leading to increased viral transcription [1]. In accordance, studies of HIV-1 C/EBP site variation show that the HIV-LTR also evolves to a high affinity C/EBPβ binding site and thus increased HIV-1 transcription [23], [24].

With regards to the observation that the +123A/G and +146C/A SNPs occur together and therefore termed “linked”, based on TESS (Transcription Element Search Site)/MatInspector analysis comparing the wild-type to +123A/G sequences no differences were found. In addition this SNP was found within the transactivation response (TAR) element where no known transcription binding sites of relevance are present [60]. Thus we focused our functional analyses on the +146C/A SNP as it was within the DS1 C/EBP site. However, the presence of the +123A/GSNP within the TAR element that forms a secondary RNA structure following transcription is intriguing. Binding of Tat to the TAR element in conjunction with transcription elongation factor (p-TEFb), and other DNA binding proteins enhance the processivity of RNA-polymerase II [61], [62]. Association of Tat with C/EBPβ has been demonstrated in a few studies [63], [64], [65]. Thus it is possible that +123A/GSNP in coordination with the +146C/A SNP in the DS1 CEBP site could potentially have implications on stabilizing the TAR structure as well as binding of Tat and C/EBPβ to the DS1 site leading to enhanced transcription and therefore these SNPs are co-selected.

It is clear that selective replication of specific viral variants and host immune responses are intimately linked. Based on our observations, during acute infection the strong innate immune responses, particularly those mediated by IFNβ in the spleen and brain, suppress replication of virus that is predominantly wild-type. This is probably due to the presence of one high-affinity LAP binding site (JC1) and two high-affinity LIP binding sites (wild-type JC1 and DS1), within the wild-type LTR [18]. The cytokine profile changes dramatically at 10 days p.i. in the spleen, with increase in IL-6 and downregulation of IFNβ mRNA levels possibly contributing to the emergence and selective replication of the DS1C/A variant. However, in the brain such changes in the cytokine prolife do not occur until 42 days p.i. We speculate that at this time LIP:LAP ratio decreases favoring LAP; virus replication resurges and the IFNβ control, observed during acute disease, is no longer maintained [1]. Additionally, it has been demonstrated that LIP has a shorter half-life compared to LAP, which could further contribute to this selection [66]. Viral genotypes with two high affinity C/EBP sites as a result of the DS1C/A substitution (wild-type JC1 and variant DS1C/A) replicate at a higher level compared to the wild-type LTR containing virus, which is no longer the predominant replicating virus in either brain or the spleen during late stages of infection.

In conclusion, results from this study clearly show that variant virus with high levels of replication in peripheral tissues, particularly one that includes infection in macrophages, can emerge very early in infection *in vivo* and provide a source of virus for infection in the CNS. Several detailed studies have been previously carried out examining the evolution of HIV/SIV *gag, env, pol* genes and the selective pressures imposed by the adaptive immune system; however, this is the first detailed study that begins to examine the influence of the host’s innate immune system and rapid selection of virus with greater infectivity than the wild-type as a result of mutations within key transcription binding sites in the SIV-LTR. Furthermore, the early emergence of variant virus in the periphery, as demonstrated here, supports the need for early antiretroviral treatment initiation and underscores the importance of instituting antiretroviral treatment regimens that dampen excessive inflammatory responses, maintain control over viral transcription/replication and include drugs that penetrate the CNS and decrease inflammatory pathways, such as minocycline [67], [68]. This is crucial in order to mediate viral suppression and prevent emergence of mutant viruses with replicative advantage that are selected by innate immune pressures early in the disease process.

## Materials and Methods

### Virus and Animal Infection

Pigtail macaques (*M. nemestrina*) were intravenously inoculated with SIV/DeltaB670 (50, 50% animal infectious doses (AID_50_)) and SIV/17E-Fr (10,000 AID_50_) as previously described [69]. SIV/DeltaB670 is an immunosuppressive swarm that was originally isolated from a rhesus macaque and used for production of virus stocks in primary rhesus lymphocytes as previously described [70], [71], [72]. SIV/17E-Fr is a cloned recombinant virus generated as described by Flaherty et al. and propagated in CEMx174 cells [29]. Animals were euthanized at 10, 21, 42, 48, or 84 days p.i., and perfused with sterile saline to remove blood from the vasculature prior to necropsy as described by Zink et al. [37].

### Ethics Statement

All animal studies were approved by the Johns Hopkins University Institutional Animal Care and Use Committee and in accordance with the recommendations of the Weatherall Report. All animal housing and care is conducted according to the Guide for the Care and Use of Laboratory Animals and the United States Department of Agriculture Animal Welfare Act. Early endpoints are adopted for this study where we aggressively monitor blood-work parameters and once progressive disease is noted the animal is humanely euthanatized. In most cases the animals in our studies are terminated at specific time points. In studies where animals may develop clinical signs, animals are euthanized if any two of the following occurs: weight loss of greater than 15% compared to baseline; CD4+ cell counts less than 5% of baseline; clinical signs of specific system disease (CNS, lung, heart); intractable diarrhea; or opportunistic infection. For the majority of our studies, nothing painful is done to the animals. Should a potentially painful procedure be performed (e.g. surgical biopsy) then animals are treated with appropriate analgesics (e.g. buprenorphine) during the procedure and periodically afterward. Procedures that could potentially be painful are always done under deep surgical anesthesia with inhaled isoflurane. All animals receive environmental enrichment e.g.: manipulanda, foraging, varied food supplements and opportunity to exhibit species-specific behavior. In terms of housing, experimental groups of 3 animals are housed as a social group prior to infection. The social group may be kept together if the study does not involve individual drug dosing, where animals are housed singly to make sure that the animals gets the appropriate drug dosage and can be monitored more closely. Even when housed individually animals are housed in cages with wire dividers so that they can still interact with their conspecifics.

### Combined Immunohistochemistry and in situ Hybridization

For double labeling tissues using immunohistochemistry and *in situ* hybridization, fixed, paraffin-embedded tissue sections were first deparaffinized by heating at 60°C, then hydrated through a graded alcohol series. Slides were then pretreated with 25 µg/mL of proteinase K (Roche Applied Science, Indianapolis, IN), washed and post-fixed with 4% paraformaldehyde (Sigma-Aldrich, St. Louis, MO). *In situ* hybridization was performed using either a sense or antisense SIVmac239 digoxigenin-UTP-labeled riboprobe at a concentration of 1.75 ng/µL overnight at 51°C. After washing, anti-digoxigenin antibody was applied for 1 h at 37°C followed by the addition of color substrate solution to slides overnight at 4°C. Uninfected and SIV-infected tissues were used as controls. After washing, slides were then blocked with 3% peroxide in methanol for 10 mins, then with normal horse serum for 20 mins followed by incubation with anti-CD68 monoclonal antibody (Dako M0814) at a concentration of 54 mg/mL for 1 h at room temperature. After washing, secondary anti-mouse IgG1k antibody was added for 30 mins following which slides were washed and treated with avidin- biotin complex (Vectastain, Vector Laboratories, Burlingame, CA) using peroxidase as the substrate for color reaction with Vector DAB (Vector) as substrate. Appropriate isotype tissue controls were used.

The total number of SIV RNA-positive macrophages in each spleen was determined by first manually counting double-labeled cells in a single cross section of spleen. The area of that piece of tissue was measured using Adobe Photoshop. The number of lymphocytes and non-CD68-positive macrophages was counted over a 3 mm^2^ area of spleen using Nikon Elements to enumerate the number of SIV RNA-positive, CD68-negative cells/cm^2^. Seven, 10 days p.i. animals (A4P020, A4P033, A4P019, A4P026, A4P027, 59T, and 616) and eight 84 days p.i. animals (Aj2, Cf2, Nl2, We2, Yv1, 72T, 389 and 01P006) were used for these studies.

### 3′RACE and PCR

RNA and DNA were extracted from brain and spleen homogenates as previously described [39]. To amplify the 3′U3-R region from tissue RNA, 3′- rapid amplification of cDNA ends (RACE) was performed on RNA serially diluted to have an equivalent of 10^5^–10^6^ copies of viral RNA as determined by real-time PCR (5 µg of total RNA was used as template when viral RNA copies were low in order to ensure cDNA synthesis). 3′RACE was conducted according to manufacturer’s protocol (Invitrogen). In brief, 200 U/ml of Superscript III reverse transcriptase (Invitrogen) was used for cDNA synthesis, together with the SuperScript™ III RT Module: 5′GCTGTCAACGATACGCTACGTAACGGCATGACAGTG(T)_24_-3′ (60 bases; Invitrogen), followed by RNase H (Invitrogen) treatment at 37°C for 20 min. The synthesized cDNA (1–2 µl) was then used as a template for two rounds of PCR. *Nef*-1 forward primer (5′GAGGCCAAAAGTTCCCCTAA-3′) and a manufacturer-provided reverse primer (GeneRacer 3′ Primer) were used for the first round of PCR, followed by nested PCR using the *nef*-2 forward primer (5′AAGAAAAGGGGGGACTGGAAGG-3′) and manufacturer-provided reverse primer (GeneRacer™ 3′Nested Primer). The forward primers complementary to the *nef* gene used for PCR were designed to span a highly conserved region of *nef* based on the *nef* sequence alignments in the Los Alamos HIV sequence database [http://www.hiv.lanl.gov/].

To amplify the 3′-LTRs from brain DNA, the DNA was either serially diluted to 10^5^–10^6^ copies of viral DNA, as measured by real-time PCR. In cases where copies of proviral DNA were low, 100 ng of total DNA was used to ensure amplification of viral LTRs. The *Nef-*1 forward and *U5*-1 reverse primer (5′-CAGGGTCTTCTTATTATTGAGTACCG-3′) were used for the first round of PCR. Nested PCR was then conducted using the *nef*-2 forward primer and the *U5*-2 reverse primer (5′- AGGAGAGATGGGAACACACA-3′). PCR amplifications were done using Platinum *Taq* DNA polymerase (Invitrogen).

### Cloning and Sequencing

PCR products were gel-purified using the Qiaquick gel extraction kit (Qiagen) and cloned into the pCR2.1-TOPO vector according to the manufacturer’s protocol (Invitrogen). An average of 96 colonies from each specified tissue and animal analyzed were sequenced using M13 forward and reverse primers (Agencourt Biosciences and Genewiz). Sequence analysis was carried out using Sequencher 4.8 software (Gene Codes Corporation, Ann Harbor, MI).

### Cell Culture

HEK-293T (human embryonic kidney) cells, obtained from American Type Culture Collection (Manassas, VA), were maintained in 10% fetal bovine serum (FBS), 10 mM HEPES, 2 mM L-glutamine, and 0.5 mg/ml gentamicin supplemented DMEM (Invitrogen Life Technologies). LuSIV cells were cultured as previously described [44].

### Plasmids

The QuikChange site-directed mutagenesis kit (Stratagene, La Jolla, CA, USA) was used to introduce the +123 A-to-G and +146 bp C-to-A (DS1C/A) in the 5′ and 3′ LTR of the SIV/17E-Fr-pUC19 viral plasmid as previously described [18]. 5′-GCTGGGCAGAGTG**G**CTCCACGCTTGC-3′ forward and 5′GCAAGCGTGGAG**C**CACTCTGCCCAGC-3′ reverse primers were used to introduce the alteration at +123 bp, while 5′-GCTTGCTTGCTTAAAG**A**CCT-3′ forward and 5′-AGG**T**CTTTAAGCAAGCAAGC-3′ reverse primers were used to introduce the + 146 bp alteration (nucleotides that are underlined and in bold indicate the specific mutations introduced). All plasmid constructs were sequenced to confirm the incorporation of mutations and the correct insertion of fragments. The expression vectors for pCMV-LAP, pCMV-FLAG-LIP and SIV/17E-Fr-pUC19 viral plasmid have been described elsewhere [13], [18], [29].

### Virus Stocks and Viral Infectivity Assay

HEK-293T cells (10^8^ cells) were seeded in T-150 flasks and transfected with Lipofectamine-2000 (Invitrogen) using 24 µg of wild-type (SIV/17E-Fr) or DS1C/A viral DNA. Supernatant was harvested 24 h post-tranfection and used to pellet virus using 20%sucrose/25 mM Tris (pH8.0), 150 mM NaCl, and 2 mM EDTA (TNE) in a Sorvall centrifuge at 26,000 rpm for 2 h at 4°C. The virus pellet was resuspended in RPMI 1640 (Invitrogen Life Technologies) supplemented with 2% FBS. For viral infectivity assays, LuSIV cells were infected with wild-type or mutant virus, as previously described using a standard input of 20 ng of SIV p27 measured by ELISA (SIV p27 ELISA kit, Zeptomatrix) [44]. Cells were treated with or without 1000 U/mL of IFNβ 1a (PBL biosciences) 4 h postinfection. Luciferase assay was carried out at 24 or 48 h postinfection as indicated in figures using the One Glo-Luciferase Reporter Assay kit (Promega Corperation) and Fluoroskan Ascent FL luminometer according to the manufacturer’s protocol. Samples were assayed in triplicate for firefly luciferase activity.

### Transient Transfection

HEK-293T cells were transfected with Lipofectamine-2000 according to manufacturer’s instructions (Invitrogen Life Techonolgies), briefly, 1×10^6^ HEK-293T cells were transfected with 4 µg of DNA (pCMV-LAP or pCMV-FLAG-LIP). Nuclear extracts were prepared at 24 h post-transfection using a nuclear extraction kit (Marligen Biosciences Inc) according to manufacturer’s protocol.

### Electrophoretic Mobility Shift Assays

The double-stranded oligonucleotide probes used in EMSAs were as follows: canonical C/EBP (5′-TGCAGATTGCGCAATCTGCA-3′); DS1 wild-type (WT) C/EBP (5-GCTTGCTTGCTTAAAGCCCT-3′); and DS1C/A (5′GCTTGCTTGCTTAAAG**A**CCT-3′). Nuclear extracts (8 µg) from HEK-293T cells transfected with pCMV-LAP or pCMV-Flag-LIP were incubated with 5×10^4^ counts/min (cpm) of [γ-^32^P] ATP-radiolabeled oligonucleotides prepared as previously described [18], [73]. Incubation was carried out in binding reaction buffer containing 20 mM HEPES (pH 8.0), 1 mM MgCl_2_, 60 mM KCl, 0.1 mM EDTA, 15% glycerol, and 1 µg poly dI-dC as a non-specific competitor. Anti-C/EBPβ antibody (sc-150X; Santa Cruz Biotechnology) and anti-FLAG M2 monoclonal antibody (Sigma-Aldrich) were used for supershift assays. For competition assays, unlabeled DS1 WT and DS1C/A oligonucleotides were added to the binding reaction at the excess concentrations noted in the figures. Samples were incubated for 30 mins at room temperature and for 15 min at 4°C prior to electrophoresis at 4°C in 5% non-denaturing TBE gels (BioRad) at 155V for 2 h. Free probe was run-off the gel in order to resolve the bands.

### Quantitation of SIV gag, IL-6, IFNβ and MxA Expression in Spleen

Total RNA was isolated from spleen tissue using the RNeasy kit (Qiagen) according to manufacturer’s protocol. RNA was treated with Turbo DNase (Ambion) at 37°C for 30 mins. SIV-gag specific primers and probes were used to determine copies of SIV RNA in 1 µg of total spleen RNA by RT-PCR performed in the QIAgility (Qiagen) using Multiplex PCR mix (Qiagen) [17]. Similarly, IL-6, IFNβand MxA expression was quantitated using primers and probes previously described [1]. For normalizing cellular mRNA levels, 18S ribosomal RNA levels were measured. Quantitation of gene expression was performed by the ΔΔCt method using the QIAgility Software (Qiagen).

### Data and Statistical Analysis

Mann-Whitney U test was used to compare the number of SIV+CD68+ macrophages in spleen of macaques euthanized at 10 and 84 days p.i. as well SIV+CD68- cells at the two time points. One-way ANOVA analysis of variance with Bonferroni post-tests, to correct for multiple sampling, (GraphPad Prism version 4.0a software) was used to assess the statistical significance of differences in the number of LTRs with wild-type (WT) genotype, variants lacking the DS1C/A and those genotypes with the DS1C/A substitution in the brain and spleen at 10–21, 42, and 48–84 days p.i. For the purposes of graphing the above results and statistical analyses, the number of clones with the indicated variations were counted; however, a value of 0 was assigned when the variant was not detected. The zero value was used to determine the p value as described above. LuSIV assay figures show mean of relative luciferase/light units (RLU) from triplicate samples **±** standard error (S.E) from three or more independent experiments. Student’s two-sided t-test was used to determine statistical significance (p<0.05). Kodak MI software was used for densitometric analysis of band intensities in the EMSAs. In competition experiments, the intensity of the complexes incubated with labeled oligonucleotide alone was set to 100%, and the intensities of complexes in all other lanes were normalized to 100%. These values were then plotted as concentration of unlabeled oligonucleotide (-log[nM]) versus the percentage (%) of protein (LAP or FLAG-LIP) bound to the labeled oligonucleotide. A non-linear regression curve was fit to this plot. Standard calculations using GraphPad Prism version 4.0a software and as described previously were performed to determine binding affinities (K_D_) based on the concentration of unlabeled oligonucleotide corresponding to the logEC_50_ values obtained from the graphs, where EC_50_ is the half maximal protein binding [18].

## Supporting Information

Figure S1 **Genotype frequencies of wild-type (WT) and LTR variants detected in brain DNA at 10, 21, 42, 48 and 84 days p.i.** Bars with gray/black background indicate the presence of +123A/G and +146C/A substitutions, collectively termed DS1C/A in the LTRs sequenced. Bars with white background indicate absence of DS1C/A substitutions. (†) Indicates variants genotypes that are non-wild-type and lack the DS1C/A substitution.
(TIF)

Figure S2 **LAP and LIP bind DS1C/A C/EBP site.** EMSA conducted using nuclear extracts derived from HEK-293T cells transfected with LAP expression vector (lanes 1–6). Binding and supershift or abrogation (where the band intensity is diminished following antibody addition) of LAP containing complexes bound to canonical (lanes 1 and 2), DS1 wild-type (WT; lanes 3 and 4), and DS1C/A (lanes 5 and 6) labeled oligonucleotides, respectively, upon addition of anti-C/EBPβ antibody (β). Nuclear extracts from HEK-293T transfected with FLAG-LIP expression (lanes 7–12) demonstrated binding and supershift of FLAG-LIP-containing complexes with anti-FLAG antibody (Flg) following incubation with the canonical (lanes 7 and 8), DS1 WT (lanes 9 and 10), and DS1C/A (lanes 11 and 12) labeled oligonucleotides, respectively.
(TIF)

Table S1 **Quantitation of SIV+/CD68+ and SIV+/CD68- cells in the spleen.** *area quantitated varied because of variable sizes of the spleen sections and the need to uniformly quantitate the same subanatomic areas in each spleen section.
(DOCX)
