# Correction: Early Emergence and Selection of a SIV-LTR C/EBP Site Variant in SIV-Infected Macaques That Increases Virus Infectivity

**DOI:** 10.1371/annotation/050af66e-a935-46e6-96ec-5ac7e0fc4dfc

**Published:** 2013-03-25

**Authors:** Shruthi Ravimohan, Lucio Gama, Elizabeth L. Engle, M. Christine Zink, Janice E. Clements

In the Funding section, the grant number AI076113 from the NIH was listed incorrectly. The correct grant number is U19AI096113. 

