# Supplementary figures and images for: Early Emergence and Selection of a SIV-LTR C/EBP Site Variant in SIV-Infected Macaques That Increases Virus Infectivity
Source: PLoS One. 2012 Aug 27;7(8):e42801. doi: 10.1371/journal.pone.0042801 (PMC3428313; doi:10.1371/journal.pone.0042801)

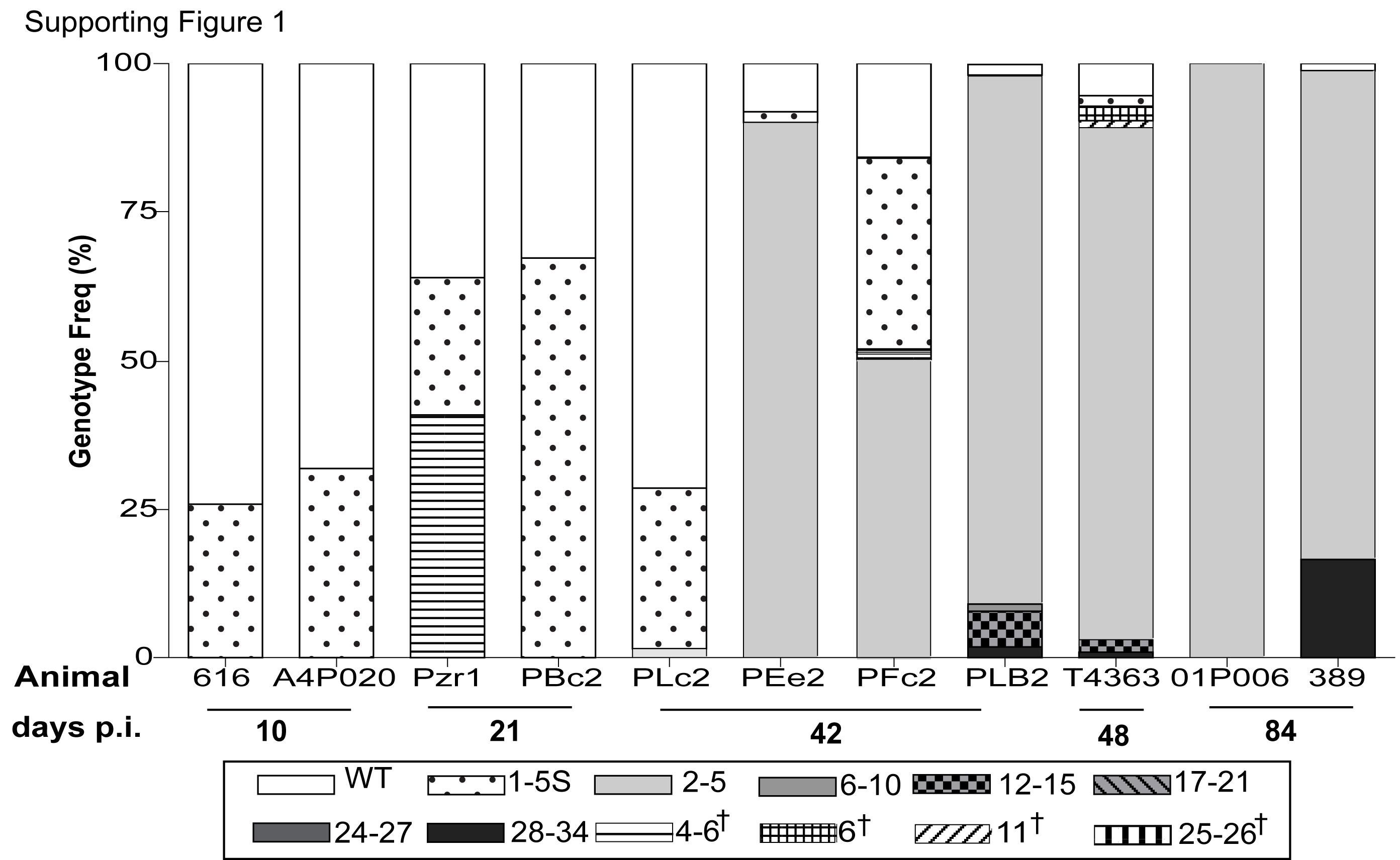

Supplement: Figure S1 — Genotype frequencies of wild-type (WT) and LTR variants detected in brain DNA at 10, 21, 42, 48 and 84 days p.i. Bars with gray/black background indicate the presence of +123A/G and +146C/A substitutions, collectively termed DS1C/A in the LTRs sequenced. Bars with white background indicate absence of DS1C/A substitutions. (†) Indicates variants genotypes that are non-wild-type and lack the DS1C/A substitution. (TIF) [file pone.0042801.s001.tif]

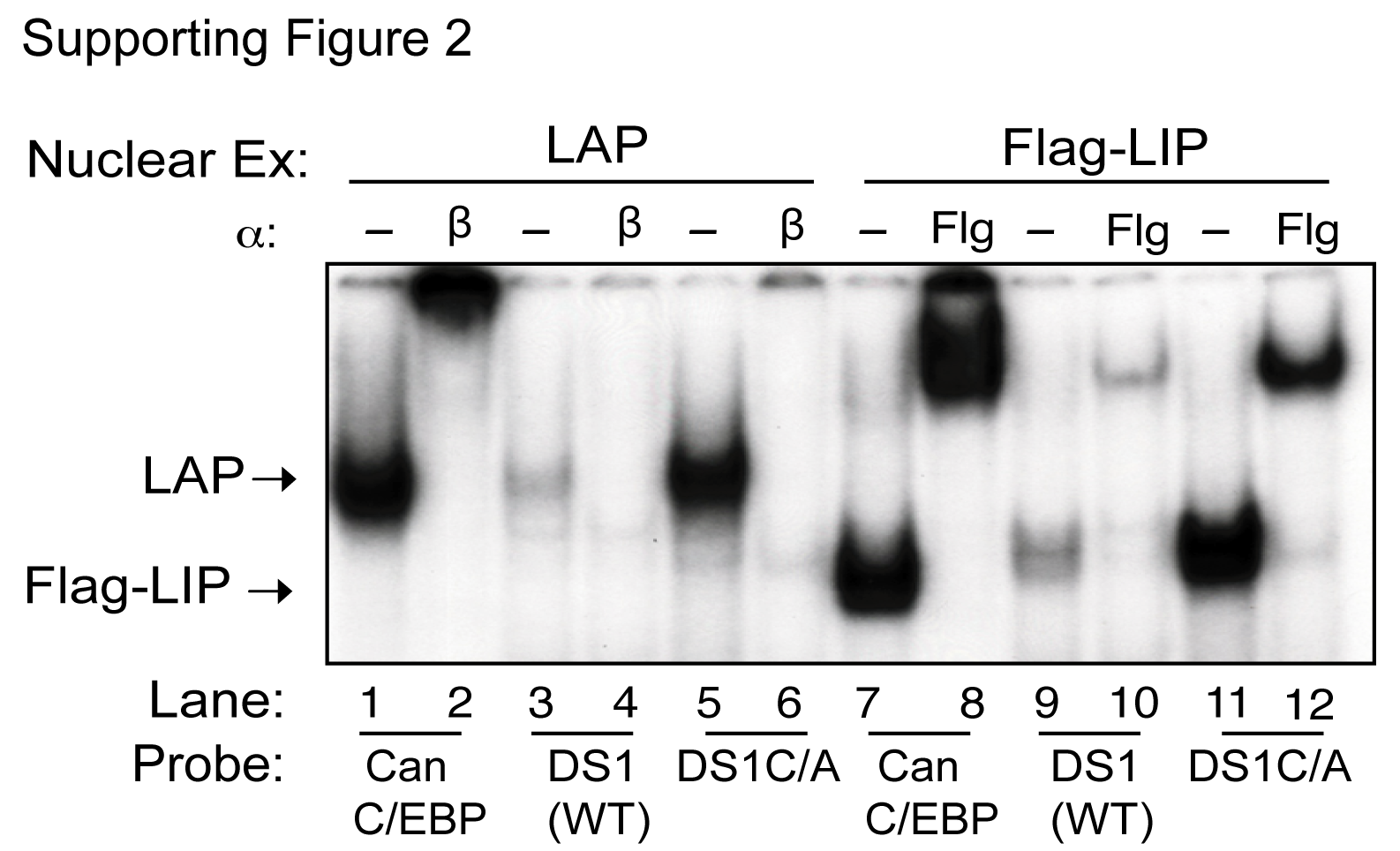

Supplement: Figure S2 — LAP and LIP bind DS1C/A C/EBP site. EMSA conducted using nuclear extracts derived from HEK-293T cells transfected with LAP expression vector (lanes 1–6). Binding and supershift or abrogation (where the band intensity is diminished following antibody addition) of LAP containing complexes bound to canonical (lanes 1 and 2), DS1 wild-type (WT; lanes 3 and 4), and DS1C/A (lanes 5 and 6) labeled oligonucleotides, respectively, upon addition of anti-C/EBPβ antibody (β). Nuclear extracts from HEK-293T transfected with FLAG-LIP expression (lanes 7–12) demonstrated binding and supershift of FLAG-LIP-containing complexes with anti-FLAG antibody (Flg) following incubation with the canonical (lanes 7 and 8), DS1 WT (lanes 9 and 10), and DS1C/A (lanes 11 and 12) labeled oligonucleotides, respectively. (TIF) [file pone.0042801.s002.tif]
