# Supplementary material for: Early Emergence and Selection of a SIV-LTR C/EBP Site Variant in SIV-Infected Macaques That Increases Virus Infectivity
Source: PLoS One. 2012 Aug 27;7(8):e42801. doi: 10.1371/journal.pone.0042801 (PMC3428313; doi:10.1371/journal.pone.0042801)
Supplement: Table S1 — Quantitation of SIV+/CD68+ and SIV+/CD68- cells in the spleen. *area quantitated varied because of variable sizes of the spleen sections and the need to uniformly quantitate the same subanatomic areas in each spleen section. (DOCX) [file pone.0042801.s003.docx]

**Table S1. Quantitation of SIV+/CD68+ and SIV+/CD68- cells in the spleen**

|  | **Monkey ID** | **Area Quantitated (cm^2^)*** | **No. SIV+/CD68+ cells/cm^2^** | **No. SIV+/CD68- cells/cm^2^** |
| --- | --- | --- | --- | --- |
| 10 days p.i. | A4P020 | 1.6 | 3.2 | 38 |
|  | A4P033 | 1.5 | 2.0 | 183 |
|  | A4P019 | 1.3 | 5.5 | 3079 |
|  | A4P026 | 1.5 | 2.4 | 287 |
|  | A4P027 | 1.1 | 0.0 | 4 |
|  | 59T | 0.7 | 23.4 | 7445 |
|  | 616 | 0.4 | 2 | 180 |
| 84 days p.i. | Aj2 | 1.6 | 0.0 | 800 |
|  | Cf2 | 1.3 | 48.9 | 4008 |
|  | Nl2 | 0.7 | 85.8 | 5635 |
|  | We2 | 1.1 | 26.4 | 9549 |
|  | Yv1 | 1.5 | 27.2 | 8187 |
|  | 72T | 1.3 | 19.3 | 8226 |
|  | 389 | 0.5 | 231 | 8920 |
|  | 01P006 | 1 | 27 | 2109 |

*area quantitated varied because of variable sizes of the spleen sections and the need to uniformly quantitate the same subanatomic areas in each spleen section.
